# Isochlorogenic acid derived from stevia improves antioxidant capacity, immune function and intestinal microbiota in weaned piglets

**DOI:** 10.3389/fvets.2025.1672217

**Published:** 2025-10-24

**Authors:** Yuxin Wang, Yong Luo, Honglei Zou, Wei Gao, Bing Yu, Jun He, Weiguang Song, Yuheng Luo, Ping Zheng, Xiangbing Mao, Yueqi Xuan, Meili Xu, Jie Yu

**Affiliations:** ^1^Key Laboratory of Animal Disease-Resistant Nutrition, Ministry of Education, Animal Nutrition Institute, Sichuan Agricultural University, Chengdu, China; ^2^Departments of Traditional Chinese Medicine, Sichuan Academy of Medical Sciences, Provincial People’s Hospital, Chengdu, China; ^3^Chenguang Biological Technology Group Co, Ltd., Handan, China; ^4^Hebei Natural Pigment Technology Innovation Center, Handan, China

**Keywords:** isochlorogenic acid, antioxidant capacity, immune function, intestinal microbiota, weaned piglet

## Abstract

Isochlorogenic acid (ICGA), a phenolic compound with demonstrated antioxidant, antibacterial, and anti-inflammatory properties, is widely present in plants. This study investigated the effects of dietary ICGA supplementation on growth performance, diarrhea incidence, antioxidant status, immune function, and intestinal microbiota in weaned piglets. A total of 180 crossbred piglets (Duroc × Landrace × Yorkshire) with an average initial body weight of 6.77 ± 0.18 kg were randomly allocated to five dietary treatments based on gender and weight. The diets consisted of a basal formulation supplemented with 0 (CON), 100, 200, 400, or 800 mg/kg ICGA for 28 days. Each treatment comprised six replicates, with six piglets per pen. Supplementation with 200 mg/kg ICGA significantly increased the average daily gain (ADG) by 3.49% during days 15–28 compared to the CON group (*p* < 0.05). Furthermore, diets containing 200 and 400 mg/kg ICGA improved the apparent total tract digestibility (ATTD) of dry matter (by 1.84 and 1.54%), crude protein (by 4.48 and 4.39%), gross energy (by 3.01 and 2.99%), ether extract (by 23.18 and 17.49%), and ash (by 8.80 and 5.13%) (*p* < 0.01). On day 14, serum catalase (CAT) activity increased by 47.78% in the 400 mg/kg group (*p* < 0.05), and this increase reached 77.65% by day 28 (*p* < 0.05). Meanwhile, the 200 mg/kg group exhibited a 75.78% elevation in total antioxidant capacity (T-AOC) on day 28 (*p* < 0.05). Serum immunoglobulin levels were also enhanced; 200 and 400 mg/kg ICGA up-regulated IgA by 23.77 and 33.42%, and IgM by 18.81 and 30.86% on day 14 (*p* < 0.01). Microbiota analysis indicated that ICGA supplementation increased the abundance of beneficial *Bacteroidota* and *Prevotella*, while reducing pathogenic taxa such as *Peptostreptococcaceae*, *Proteobacteria*, and *Staphylococcus*. In conclusion, dietary ICGA at 200–400 mg/kg effectively reduced diarrhea incidence, enhanced nutrient digestibility, improved antioxidant capacity, strengthened humoral immunity, and positively modulated gut microbiota in weaned piglets. Further research is warranted to elucidate the underlying mechanisms and assess the potential for practical application in swine production.

## 1 Introduction

Weaning is a critical challenge in pig production, as multiple stressors compromise piglet health and growth. Abrupt separation from the sow, dietary shift from milk to solid feed, and environmental alterations frequently trigger post-weaning syndrome, manifested as reduced feed intake, intestinal inflammation, oxidative stress, and heightened susceptibility to enteric infections. These disruptions lead to growth retardation, impaired nutrient utilization, and increased morbidity and mortality, causing substantial economic losses. Although antibiotic growth promoters have been widely employed to enhance growth and prevent disease, rising concerns regarding antimicrobial resistance have driven the pursuit of sustainable plant-based alternatives that support gastrointestinal health and immune function without relying on conventional antimicrobials.

Isochlorogenic acid (ICGA), a structural analog of chlorogenic acid (CGA), is a naturally occurring polyphenolic compound synthesized via the shikimic acid pathway during aerobic respiration in plants (1). The shikimic acid pathway, a key metabolic route in plants and microorganisms, produces aromatic amino acids and secondary metabolites. It initiates with the condensation of phosphoenolpyruvate and erythrose-4-phosphate, ultimately generating precursors for numerous phenolic compounds like ICGA. ICGA is found in a variety of plant species, including *Ilex hainanensis*, *Atractylodes macrocephala*, *Stevia rebaudiana*, and *Lonicera periclymenum* (2, 3). This compound exists as three distinct isomers, ICGA A, B, and C, which differ in their molecular structures (molecular formula: C_25_H_24_O_12_; molecular weight: 516.45) (4). In contrast to CGA, ICGA contains one molecule of quinic acid linked to two molecules of caffeic acid, making it structurally more complex by having an additional caffeic acid group (5, 6). Research has demonstrated that ICGA exhibits a broad spectrum of biological activities, including antioxidant, antibacterial, antiviral, anti-inflammatory, hepatoprotective, and neuroprotective effects, highlighting its therapeutic potential (5, 7–10). Additionally, ICGA is included as an active ingredient in several established pharmaceutical formulations, such as Siji-kangbingdu Mixture, Shuanghuanglian Granules, and Reduning Injection, underscoring its relevance in both traditional and modern medicinal applications (5). Given its diverse biological activities, ICGA presents a promising alternative to conventional antibiotic feed additives in the livestock and poultry industries, offering a potential strategy for promoting animal health while reducing reliance on antibiotics.

CGA has garnered significant attention as a promising alternative to antibiotics in livestock production, with growing evidence supporting its beneficial effects on animal health, especially in pigs, CGA is also known to alleviate intestinal damage, ameliorate oxidative stress, and regulate mitochondrial function (11, 12). However, despite their structural similarities, the biological and pharmacological properties of ICGA may differ from those of CGA due to variations in their chemical configurations. While the antimicrobial mechanisms of CGA, including bacterial injury repair, are well documented (13), few studies have examined ICGA’s effects in pigs. Recent research on other plant-derived supplements supports the potential of phytogenic additives. For example, *Litsea cubeba* essential oil enhanced growth performance, immunity, antioxidant status, nutrient digestibility, and fecal microflora in pigs (14). Similarly, rumen-protected lysine supplementation improved nitrogen utilization and modified hindgut microbiota in dairy cows (15), illustrating how targeted dietary interventions can modulate gut health. To address the lack of ICGA-specific studies, we evaluated the effects of dietary ICGA supplementation on growth performance, antioxidant status, immune function, and gut microbiota in weaned piglets. This study aims to elucidate ICGA’s potential as a novel feed additive for sustainable livestock production.

## 2 Materials and methods

### 2.1 Experimental animals, diet, design and housing

The ICGA used in this experiment was sodium isochlorogenic acid (Chenguang Biotechnology Group Co., Ltd., Hebei, China, batch number: 2-0969-200613), which was derived from stevia (*Stevia rebaudiana*) through a standardized extraction process. The active ingredient, ICGA content in this brownish-yellow powder was 51.3%. The animal care and experimental procedures were approved by the Institutional Animal Care and Use Committee of Sichuan Agricultural University (No. 20190129).

In this experiment, 180 weaned piglets (Duroc × Landrace × Yorkshire; 21 d of age; initial BW = 6.77 ± 0.18 kg) were allotted to 5 dietary treatments. Each treatment comprised 6 replicate pens with 6 piglets per pen (3 barrows and 3 gilts). The basal diet was supplemented with 0 (CON), 100, 200, 400, or 800 mg/kg of ICGA. We selected this dose range based on the efficacy of its structural analog, CGA, which improves growth performance and intestinal health in weaned pigs at 500–1000 mg/kg (16). The 100–800 mg/kg gradient was designed to identify the optimal and potentially lower effective dose of ICGA, considering its distinct bioavailability and efficacy. The 28-day experimental period encompassed the critical post-weaning recovery and growth phase. This duration allowed sufficient time for significant differences in average daily gain (ADG) and feed efficiency (G: F) to manifest and is consistent with established methodologies for assessing the medium-term effects of dietary additives in swine. The nutrient composition of the basal diet (Table 1) met or exceeded the dietary requirements for weaned piglets as outlined by the National Research Council (NRC, 2012).

**Table 1:** Ingredients composition and nutrient levels of basal diets (%, as-fed basis).

Item	Contents	Nutrient level^1^	Contents
Corn	31.70	DE, Mcal/kg	3.53
Extruded corn	30.25	CP	18.70
Soybean meal	7.90	OM	94.68
Extruded soybean	8.50	ADF	2.91
Fish meal	3.83	NDF	7.61
Whey powder	5.40	Ca	0.78
Soybean protein concentrate	6.60	Total P	0.57
Soybean oil	1.60	Available P	0.38
Glucose	2.00	D-Lysine	1.30
Limestone	0.60	D-Methionine	0.38
Dicalcium phosphate	0.50	D-Threonine	0.77
Salt	0.20	D-Tryptophan	0.22
L-Lys·HCl	0.32		
DL-Methionine	0.07		
L-Threonine	0.02		
Tryptophan	0.01		
Chloride choline	0.15		
Vitamin premix^2^	0.05		
Mineral premix^3^	0.30		
Total	100.00		

^1^Crude protein (CP), calcium (Ca), total phosphorus (P), organic matter (OM), acid detergent fiber (ADF) and neutral detergent fiber (NDF) are measured values, the rests are calculated values. ^2^The premix provides following per kilogram of diet: vitamin A, 15,000 IU; vitamin D_3_, 5,000 IU; vitamin E, 40 mg; vitamin K, 5 mg; vitamin B_1_, 5 mg; vitamin B_2_, 12.5 mg; vitamin B_6_, 6 mg; vitamin B_12_, 0.06 mg; folic acid, 2.5 mg; nicotinic acid, 50 mg; D-pantothenic acid, 25 mg; D-biotin, 0.25 mg. ^3^The premix provides following per kilogram of diet: Fe (as ferrous sulfate), 100 mg; Cu (as copper sulfate), 6 mg; Mn (as manganese sulfate), 4 mg; Zn (zinc sulfate), 100 mg; I (potassium iodide), 0.14 mg; Se (as sodium selenite), 0.30 mg.

The piglets were housed in standard flat-bed pens, each equipped with duckbill drinkers, adjustable feeders, and perforated plastic flooring. Throughout the experiment, the piglets had ad libitum access to both feed and water. The environmental conditions within the pens were carefully controlled, with a temperature range maintained between 25 to 28 °C and relative humidity held at 60 to 70%. To uphold optimal health and hygiene standards, the pens were thoroughly cleaned and disinfected daily, ensuring a safe and sterile environment for the animals throughout the study period.

### 2.2 Growth performance and diarrhea

The piglets were monitored daily to assess their health status throughout the experiment. Daily feed intake for each pen was recorded, and the average daily feed intake (ADFI) was calculated. The body weight of fasted piglets was measured on days 1, 15, and 29 of the trial to determine the average daily gain (ADG) and feed-to-gain ratio (F: G). To evaluate the incidence and severity of diarrhea, fecal consistency was scored on a 0 to 3 scale: 0 = normal, firm feces; 1 = soft feces, potential slight diarrhea; 2 = unformed, moderately fluid feces; and 3 = very watery, frothy diarrhea. Diarrhea was defined as a score of 2 or greater. The diarrhea rate and diarrhea index were calculated as follows: diarrhea rate (%) = (number of piglets with diarrhea per pen × days of diarrhea) / (total number of piglets × 28 days) × 100, and diarrhea index = sum of diarrhea scores per pen/(number of piglets per pen × total days) (17).

### 2.3 Sampling and measurements

Fecal samples were obtained on d 25 and 28 to capture any changes in nutrient utilization once piglets had adapted to their diets. Immediately after collection, each 100 g sample of fresh manure (from pens housing 6 piglets) was treated with 10 mL of 10% H_2_SO_4_ to reduce nitrogen volatilization. At the conclusion of the trial, all fecal samples from each pen were thoroughly mixed, dried at 65 °C for 96 h, and then finely ground through a 1 mm screen to ensure uniformity.

All feed and fecal samples were analyzed for dry matter (Method 930.15), crude protein (Method 990.03), ether extract (Method 920.39), and ash (Method 942.05), according to AOAC (2005). Gross energy was quantified using an adiabatic oxygen bomb calorimeter (Parr Instrument Co., Moline, IL, USA). The organic matter (OM) content in feeds and feces was calculated by subtracting the crude ash content from the dry matter (DM). Neutral detergent fiber (NDF) and acid detergent fiber (ADF) in feeds and feces was determined using Method 973.18 with an Ankom A200i fiber analyzer (Ankom Technology, Macedon, NY, USA). The analysis employed heat-stable *α*-amylase and sodium sulfite, with residual ash uncorrected. Acid-insoluble ash (AIA), a robust endogenous marker for nutrient digestibility, was determined using the procedure described by China Standards Press (2009). Apparent total digestibility (ATTD) of nutrients was then calculated based on (18), using the following equation:


$$\text{ATTD}\;(\%)=1-[\frac{\mathrm{AIA}\;\text{diet}\times \text{Nutrient feces}}{\mathrm{AIA}\;\text{feces}\times \text{Nutrient diet}}]\times 100.$$

Blood samples (10 mL) were collected on d 14 and d 28 from the anterior cava vein of one piglet per pen. The samples were drawn into vacuum tubes lacking anticoagulant and centrifuged at 3,500 × g for 10 min at 4 °C to separate the serum. Serum aliquots were immediately transferred into clean centrifuge tubes and stored at −20 °C for subsequent laboratory analyses. To further investigate local intestinal immune status, one piglet (per replicate pen) was chosen at the end of the trial and euthanized via an intravenous injection of chlorpromazine hydrochloride (3 mg/kg body weight). After opening the abdominal cavity, the jejunum, ileum, colon, and cecum were isolated according to standard anatomical markers. The contents of the ileum, colon, and cecum were aseptically gathered in sterile cryopreservation tubes for microbiological and biochemical evaluations. Additionally, approximately 10 cm of jejunum and ileum (the same segment from each pig) were dissected longitudinally, rinsed with 0.9% ice-cold saline to remove digesta, and gently scraped with a sterile microscope slide to collect the mucosal layer. Care was taken to use a new slide for each segment to reduce cross-contamination; all procedures were carried out on ice. Mucosal samples were then placed in sterile frozen storage tubes and preserved at −80 °C.

Serum antioxidant capacity was measured by determining T-AOC (catalog No. A015-1-2), CAT activity (catalog No. A007-1-1), SOD activity (catalog No. A001-1-2), GSH-Px activity (catalog No. A005-1-2), and MDA concentration (catalog No. A003-1-2). These assays followed the instructions provided by the kit manufacturer (Nanjing Jiancheng Institute of Bioengineering, Jiangsu, China). Serum concentrations of IgA (catalog No. 8101), IgG (catalog No. 528), and IgM (catalog No. 521) were quantified using ELISA kits from Jiangsu Meimian Industrial Co., Ltd. (Jiangsu, China). In parallel, intestinal mucosal samples were analyzed for sIgA (catalog No. 9505), IL-2 (catalog No. 5010), IL-4 (catalog No. 5005), IL-10 (catalog No. 5026), and IFN-*γ* (catalog No. 23114) using an ELISA kit obtained from the same supplier.

### 2.4 Analysis for microbial community by 16S rRNA sequences

Colonic digesta samples (*n* = 30) were collected from pigs assigned to five dietary treatments: control, 100 mg/kg ICGA, 200 mg/kg ICGA, 400 mg/kg ICGA, and 800 mg/kg ICGA, with six animals per group. To profile the resident microbiota, the V3–V4 hypervariable region of the bacterial 16S rRNA gene was amplified and sequenced. Sample preparation, DNA extraction and validation, PCR amplification, product purification, library construction and quality assessment, as well as high-throughput sequencing using the NovaSeq platform, were performed by Beijing Novogene Biotechnology Co., Ltd.

Raw reads were preliminarily filtered and merged to remove low-quality or chimeric sequences, resulting in a clean dataset suitable for downstream analyses. DADA2 (Version 1.8) was then used to denoise these reads, with sequences falling below a minimum abundance threshold of 5 excluded according to Li *et al* (19). The remaining ASVs served as the basis for taxonomic classification and quantification of relative abundances. Representative ASVs were annotated with species-level identifiers, facilitating both compositional and alpha diversity assessments. To further investigate potential group-specific microbial signatures, pairwise t-tests were performed on the final dataset, and LEfSe was employed to identify differentially enriched taxa among the categorized samples.

### 2.5 Statistical analysis

We collated all experimental data using Microsoft Excel 2019. Prior to statistical analysis, we assessed data normality with the Shapiro–Wilk test. We then performed a one-way ANOVA using SAS 9.4 (SAS Inst. Inc., Cary, NC). When the ANOVA indicated significant differences, we applied Duncan’s multiple range test for *post hoc* comparisons. To evaluate dose–response relationships, we conducted linear and quadratic regression analyses on the effects of dietary ICGA supplementation. Results are expressed as mean ± standard error (SE). We considered differences statistically significant at *p* < 0.05 and indicative of a trend at 0.05 ≤ *p* < 0.10. The statistical models are as follows:


$$Y_{\mathrm{ij}}=\mu +\tau_{i}+\in_{\mathrm{ij}}$$

$Y_{\mathrm{ij}}$: Observed value of the *j*-th replicate in the *i*-th treatment group. μ: Grand mean, the theoretical average of all observations. $\tau_{i}$: Fixed effect of the *i*-th treatment, representing its deviation from μ (∑$\tau_{i}$=0). $\in_{\mathrm{ij}}$: Random error term, independently and identically distributed as *N* (0,*σ*^2^).


$$Y_{i}=\beta_{0}+\beta_{1}X_{i}+\in_{i}$$

$Y_{i}$: Response value of the *i*-th observation. $\beta_{0}$: Regression intercept, the predicted Y when X=0. $\beta_{1}$: Regression slope, indicating the average change in Y per 1-unit increase in X. $X_{i}$: ICGA dose level (continuous variable, values: 0, 100, 200, 400, 800 mg/kg). $\in_{i}$: Random error term, following *N* (0, *σ*^2^).


$$Y_{i}=\beta_{0}+\beta_{1}X_{i}+\beta_{2}X_{i}^{2}+\in_{i}$$

$Y_{i}$: Response value of the *i*-th observation. $\beta_{0}$: Regression intercept, the predicted Y when X=0. $\beta_{1}$: Regression slope, indicating the average change in Y per 1-unit increase in X. $X_{i}$: ICGA dose level (continuous variable, values: 0, 100, 200, 400, 800 mg/kg). $\in_{i}$: Random error term, following *N* (0,*σ*^2^). $\beta_{2}$: Quadratic term coefficient, reflecting nonlinear (curvilinear) effects. If $\beta_{2}$≠0, a quadratic relationship exists.

## 3 Results

### 3.1 Growth performance and diarrhea rate

The effects of ICGA supplementation in the diets on growth performance, diarrhea rate and diarrhea index are presented in Table 2. From days 15 to 28, dietary supplementation with 200 mg/kg ICGA significantly increased the average daily gain (ADG) by 3.49% compared with the CON group (*p* < 0.05). The ICGA dosage also showed a quadratic regression relationship (*p* < 0.05). ICGA supplementation linearly reduced diarrhea rate of weaning piglets during days 0–14 and 0–28 (*p* < 0.05).

**Table 2:** Effects of isochlorogenic acid (ICGA) supplementation on growth performance, diarrhea rate and diarrhea index in weaned piglets.

Item	ICGA (mg/kg)					SEM	*p*-value		
	0	100	200	400	800		ANOVA	Linear	Quadratic
Initial BW, kg	6.78	6.77	6.78	6.77	6.77	0.175	1.000	0.991	1.000
Final BW, kg	12.73	12.95	13.79	13.49	12.86	0.284	0.756	0.700	0.469
0–14 d									
ADFI (g)	179.41	174.30	169.93	184.85	181.62	4.070	0.814	0.612	0.709
ADG (g)	100.04	98.32	93.92	123.94	97.33	4.081	0.128	0.509	0.740
F: G ratio	1.80	1.80	1.83	1.50	1.99	0.058	0.104	0.846	0.471
Diarrhea rate, %	17.66	17.26	16.87	14.48	8.53	1.361	0.175	0.025	0.040
Diarrhea index	0.37	0.37	0.36	0.31	0.19	0.027	0.198	0.125	0.296
15–28 d									
ADFI (g)	490.96	510.93	563.70	548.46	500.28	12.121	0.249	0.522	0.103
ADG (g)	323.50^b^	342.73^b^	411.53^a^	358.33^ab^	334.79^b^	10.347	0.049	0.611	0.043
F: G ratio	1.54	1.49	1.37	1.54	1.50	0.024	0.150	0.846	0.311
Diarrhea rate, %	16.07	11.71	11.90	10.12	10.12	1.233	0.565	0.124	0.251
Diarrhea index	0.35	0.26	0.26	0.22	0.22	0.026	0.484	0.089	0.193
0–28 d									
ADFI (g)	335.19	342.61	366.81	366.65	340.95	7.426	0.532	0.508	0.283
ADG (g)	212.98	220.53	251.24	241.06	216.23	5.813	0.147	0.520	0.071
F: G ratio	1.58	1.56	1.46	1.53	1.59	0.021	0.317	0.867	0.159
Diarrhea rate, %	16.87	14.48	14.38	12.30	9.33	1.138	0.304	0.029	0.091
Diarrhea index	0.36	0.32	0.31	0.21	0.26	0.021	0.295	0.064	0.155

ADFI, average daily feed intake; ADG, average daily gain; F: G ratio, feed-to-gain ratio. ^a,b^Within a row, means without a common superscript letter differ at *p* < 0.05.

### 3.2 Nutrient digestibility

As shown in Table 3, Relative to the CON group, pigs receiving 200 or 400 mg/kg ICGA exhibited significantly improved apparent total tract digestibility (ATTD; *p* < 0.01) of dry matter (1.84 and 1.54%, respectively), crude protein (4.48 and 4.39%), gross energy (3.01 and 2.99%), ether extract (23.18 and 17.49%), and ash (8.80 and 5.13%).

**Table 3:** Effects of isochlorogenic acid (ICGA) supplementation on nutrient digestibility in weaned piglets (%).

Item	ICGA (mg/kg)					SEM	*P*-value		
	0	100	200	400	800		ANOVA	Linear	Quadratic
DM CP GE EE Ash	92.93^c^ 77.22^b^ 84.72^b^ 74.09^b^ 45.68^b^	93.57^bc^ 76.36^b^ 84.25^b^ 70.07^c^ 47.30^b^	94.64^a^ 80.68^a^ 87.27^a^ 80.61^a^ 56.27^a^	94.36^ab^ 80.61^a^ 87.25^a^ 77.89^a^ 53.67^a^	93.32^c^ 81.34^a^ 87.78^a^ 77.92^a^ 54.95^a^	0.172 0.502 0.320 0.832 0.929	0.002 <0.001 <0.001 <0.001 <0.001	1.000 0.271 0.150 0.980 0.064	0.010 0.520 0.334 0.771 0.050

DM, dry matter; CP, crude protein; GE, gross energy; EE, ether extract. ^a–c^Within a row, means without a common superscript letter differ at *P* < 0.05.

### 3.3 Serum anti-oxidative properties

The effects of dietary ICGA supplementation on serum anti-oxidation are shown in Table 4. On day 14, the 400 mg/kg ICGA group showed a 47.78% increase in serum catalase (CAT) activity compared with the CON group (*p* < 0.05), which further increased to 77.65% by day 28 (*p* < 0.05). Meanwhile, supplementation with 200 mg/kg ICGA elevated serum total antioxidant capacity (T-AOC) by 75.78% on day 28 (*p* < 0.05), demonstrating a significant quadratic dose–response relationship (*p* < 0.05).

**Table 4:** Effects of isochlorogenic acid (ICGA) supplementation on antioxidant index in serum of piglets.

Item	ICGA (mg/kg)					SEM	*P*-value		
	0	100	200	400	800		ANOVA	Linear	Quadratic
Day 14									
T-AOC, U/ml	0.83	1.28	1.50	1.15	1.24	0.103	0.338	0.587	0.460
CAT, U/ml	10.63^b^	11.34^b^	11.27^b^	15.71^a^	11.07^b^	0.616	0.042	0.244	0.467
SOD, U/ml	190.05	189.64	180.57	190.17	191.65	2.468	0.655	0.884	0.947
GSH-Px, U/ml	444.28	433.61	490.83	454.66	438.81	9.047	0.287	0.833	0.425
MDA, nmol/ml	4.59	4.68	4.04	3.80	3.65	0.219	0.496	0.221	0.413
Day 28									
T-AOC, U/ml	1.28^b^	1.74^ab^	2.25^a^	1.65^ab^	1.22^b^	0.121	0.020	0.438	0.040
CAT, U/ml	10.38^b^	10.15^b^	11.23^b^	18.44^a^	7.59^b^	0.989	0.003	0.960	0.576
SOD, U/ml	213.18	198.88	209.25	203.04	204.70	2.654	0.504	0.831	0.507
GSH-Px, U/ml	336.85	351.59	303.78	320.49	327.25	8.290	0.492	0.974	0.955
MDA, nmol/ml	5.39	4.86	4.74	4.48	4.77	0.186	0.634	0.347	0.326

T-AOC, total antioxidant capacity; CAT, catalase; SOD, total superoxide. Dismutase; GSH-Px, glutathione peroxidase; MDA, malondialdehyde. ^a,b^Within a row, means without a common superscript letter differ at *P* < 0.05.

### 3.4 Immunoglobulins in serum, cytokines in intestinal mucosa and sIgA

The effects of dietary ICGA supplementation on immunoglobulins in serum are shown in Table 5. At day 14, serum immunoglobulin A (IgA) levels increased by 23.77 and 33.42%, and immunoglobulin M (IgM) by 18.81 and 30.86%, in the 200 and 400 mg/kg ICGA groups, respectively (*p* < 0.01). Immunoglobulin levels exhibited a significant quadratic relationship with ICGA dose (*p* < 0.01), peaking at 400 mg/kg. The effects of dietary ICGA supplementation on cytokines in intestinal mucosa are shown in Table 6. Obviously, no differences were observed for sIgA, IL-2, IL-4, IL-10, and IFN-*γ* in ileal and jejunal mucosa of weaned piglets among the 5 dietary treatments (*p* > 0.05).

**Table 5:** Effects of isochlorogenic acid (ICGA) supplementation on immunoglobulins in serum of piglets.

Item	ICGA (mg/kg)					SEM	*P*-value		
	0	100	200	400	800		ANOVA	Linear	Quadratic
Day 14									
IgA, μg/ml	25.58^c^	29.39^b^	31.66^ab^	34.13^a^	32.67^ab^	0.727	<0.001	<0.001	<0.001
IgG, μg/ml	299.07	338.29	356.55	361.14	343.80	7.810	0.082	0.040	0.014
IgM, μg/ml	33.34^c^	36.87^bc^	39.61^ab^	43.63^a^	40.87^ab^	1.052	0.013	0.002	0.003
Day 28									
IgA, μg/ml	29.88	29.07	36.14	32.18	30.73	0.892	0.086	0.455	0.195
IgG, μg/ml	295.78	330.06	333.23	325.28	294.36	7.210	0.233	0.884	0.058
IgM, μg/ml	35.47^bc^	37.29^bc^	34.60^c^	39.63^b^	47.48^a^	1.033	<0.001	<0.001	<0.001

IgA, immunoglobulin A; IgG, immunoglobulin G; IgM, immunoglobulin M. ^a–c^Within a row, means without a common superscript letter differ at *P* < 0.05.

**Table 6:** Effects of isochlorogenic acid (ICGA) supplementation on sIgA and cytokines levels in intestinal mucosa of piglets.

Item	ICGA (mg/kg)					SEM	*P*-value		
	0	100	200	400	800		ANOVA	Linear	Quadratic
Ileum									
sIgA, μg/ml	36.01	35.57	38.04	42.25	40.03	2.064	0.853	0.322	0.615
IL-2, pg/ml	446.93	451.53	469.45	485.46	499.45	14.812	0.797	0.189	0.425
IL-4, ng/L	92.62	100.39	92.18	94.27	109.59	6.727	0.929	0.568	0.774
IL-10, ng/ml	177.00	170.40	171.74	171.65	196.63	8.536	0.875	0.512	0.566
IFN-γ, pg/ml	2975.04	2975.49	2843.91	2949.33	3063.89	250.768	0.999	0.934	0.974
Jejunum									
sIgA, μg/ml	40.88	43.14	44.40	37.69	42.92	2.083	0.879	0.569	0.850
IL-2, pg/ml	541.50	525.49	526.12	490.86	538.12	15.572	0.857	0.931	0.919
IL-4, ng/L	155.39	140.18	157.50	141.27	150.83	8.409	0.960	0.660	0.693
IL-10, ng/ml	222.07	205.70	235.10	176.54	214.89	11.322	0.561	0.798	0.916
IFN-γ, pg/ml	4943.55	4892.39	4882.52	3920.02	4784.37	388.362	0.915	0.831	0.916

sIgA, secretory immunoglobulin A; IL-2, interleukin-2; IL-4, interleukin-4; IL-10, interleukin-10; IFN-γ, interferon-γ.

### 3.5 Microflora community

The change in bacterial diversity was investigated using the 16 s rRNA sequencing. The alpha diversity analysis index (observed_otus, shannon, simpson, chao1, and goods_coverage) of each sample is counted. As shown in Table 7, there were no statistical differences in the alpha diversity of colonic digesta microbial communities among the 5 treatments.

**Table 7:** Effects of isochlorogenic acid (ICGA) supplementation on the α-diversity^1^ of microbial communities of weaning piglets in colonic digesta.

Item	ICGA (mg/kg)					SEM	*P*-value		
	0	100	200	400	800		ANOVA	Linear	Quadratic
Observed_otus	798.25	686.00	629.33	691.60	568.67	26.130	0.081	0.016	0.053
Chao^1^	826.34	703.34	657.97	718.66	598.61	27.531	0.132	0.029	0.087
Shannon	7.55	7.63	7.15	7.42	6.80	0.106	0.078	0.020	0.055
Simpson	0.98	0.99	0.97	0.98	0.97	0.003	0.366	0.272	0.523
Goods_coverage	0.99	0.99	0.99	0.99	0.99	0.001	0.890	0.996	0.957

^1^Alpha-diversity analysis for bacterial community determined by 16S rRNA gene sequencing.

**Figure 1:**
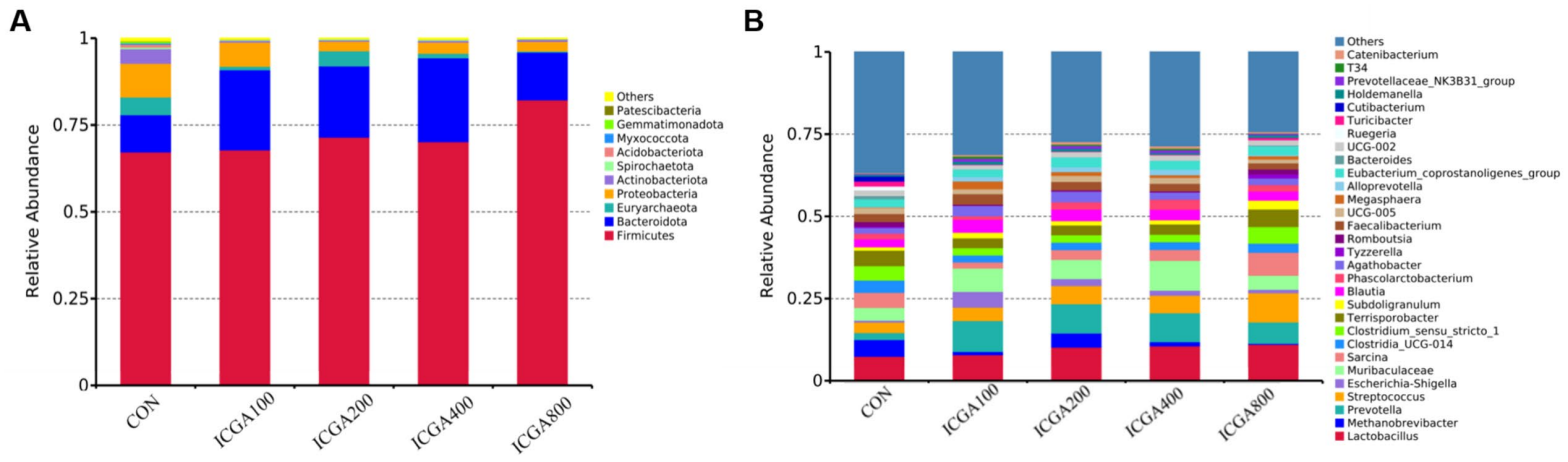
The relative abundance of top 10 microbial community bar plot on the phylum and genus level. Values are the means (*n* = 6 replicates per treatment). **(A)** The relative abundance of top 10 microbial community bar plot on the phylum level. **(B)** The relative abundance of top 30 microbial community bar plot on the genus level.

**Figure 2:**
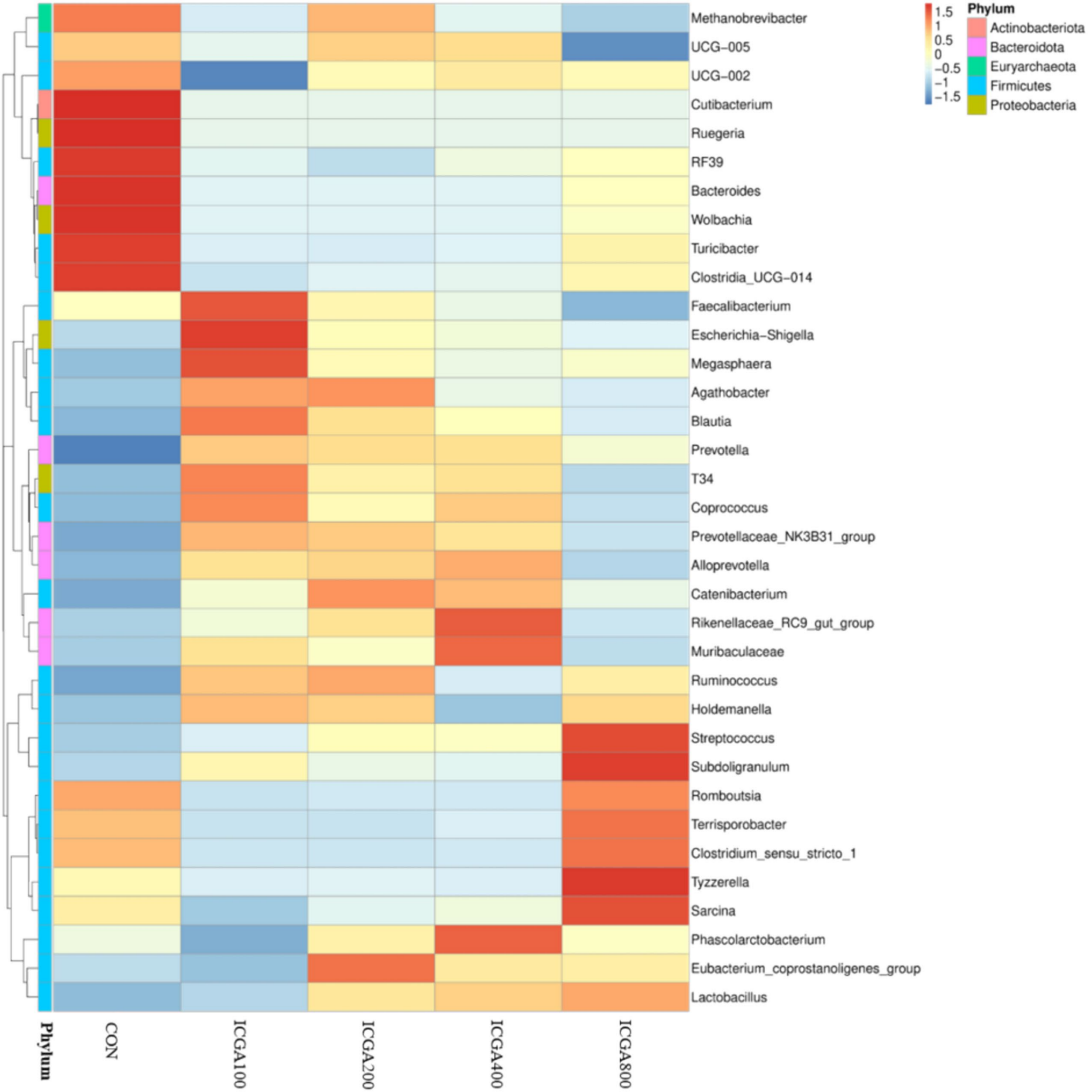
Heatmap of top 35 genera (relative abundances) among the groups. Values are the means (*n* = 6 replicates per treatment).

The histogram of relative abundance of species can not only display the dominant species and composition of each sample, but also clearly observe the change trend of the abundance of dominant species in different species. At the phylum level, Firmicutes, Bacteroidota, Actinobacteriota and Proteobacteria were the dominant microbial divisions (Figure 1A). At the genus level, *Lactobacillus*, *Methanobrevibacter*, *Prevotella*, *Streptococcus*, *Muribaculaceae*, *Sarcina*, *Clostridia_UCG-014*, *Clostridium_sensu_stricto_1*, *Terrisporobacter*, *Blautia*, *Faecalibacterium*, and *Eubacterium_coprostanoligenes_group* were predominant (Figure 1B). The heatmap plot (according to the top 35, the most different genera) showed the relative abundance of genera in different groups (Figure 2). The color gradient and similarity degree on the heatmap plot reflected the similarity and difference of community composition among multiple samples.

**Figure 3:**
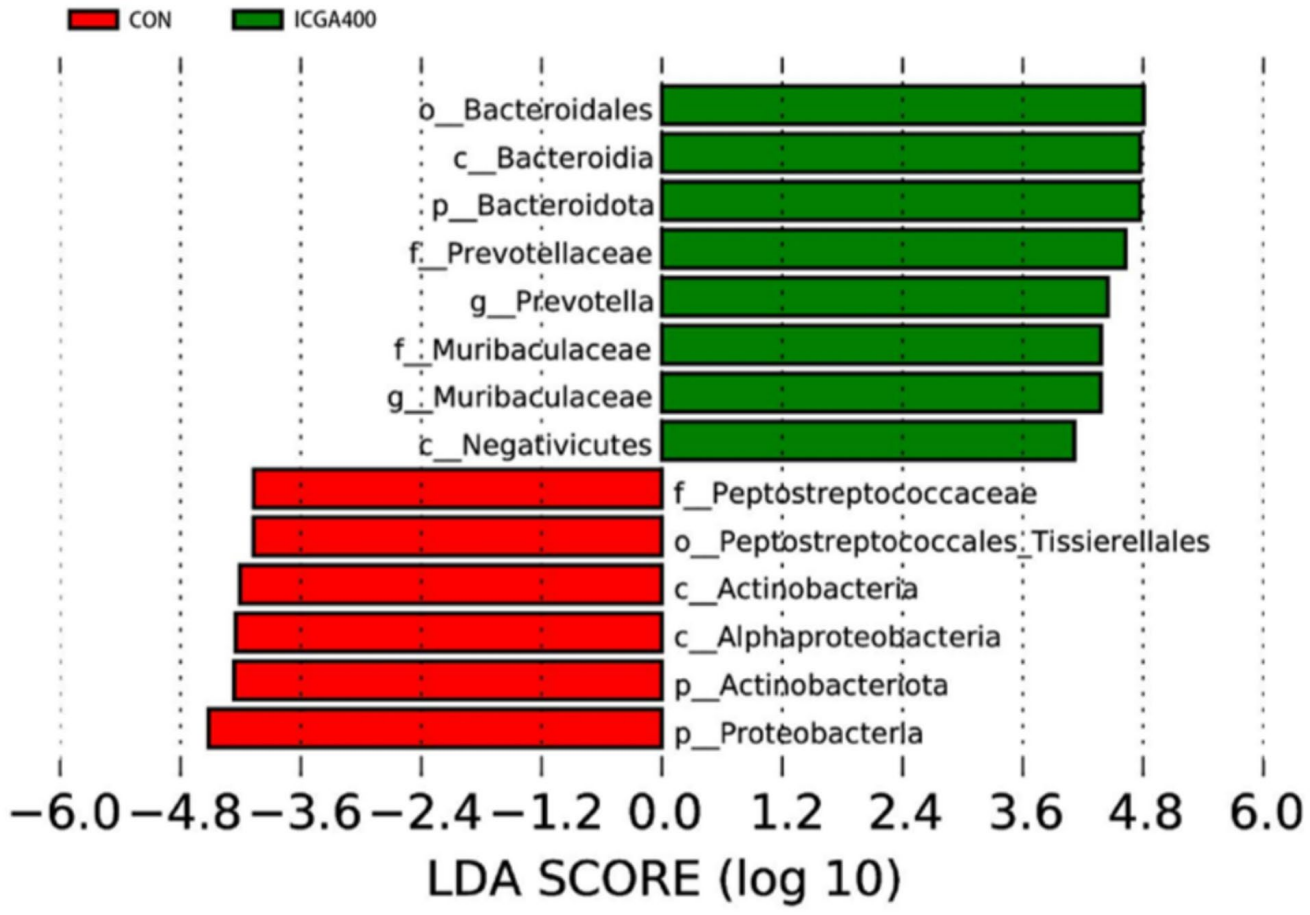
Linear discriminant analysis (LDA) scores (>4.0) computed for features at the ASV level. Letters represented the taxonomy of the bacteria: p, phylum, c, class; o, order; f, family; g, genus. All the values contained 6 repetitions. ICGA400 = 400 mg/kg isochlorogenic acid. Values are the means (*n* = 6 replicates per treatment).

LEfSe analysis was used to study the influence degree of species that were significantly different (LDA score > 4.0) from phylum to genus level, and results included LDA value distribution histogram. (Figure 3). We found that Bacteroidota, *Prevotella*, and *Muribaculaceae* were the dominant species in the ICGA400 group. *Peptostreptococcaceae*, Actinobacteriota, and Proteobacteria were the main components of gut microbiota in the CON group.

**Figure 4:**
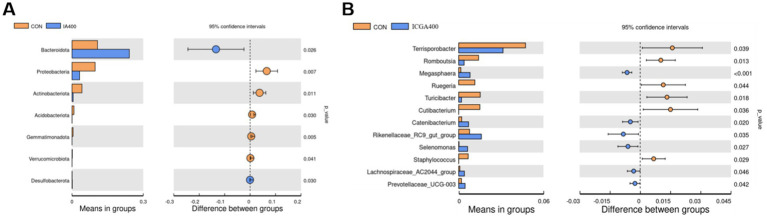
The t-test bar plot between groups. **(A)** The *t*-test bar plot with significantly different phylum between groups. **(B)** The *t*-test bar plot with significantly different genera between groups. Values are the means (*n* = 6 replicates per treatment).

T-test can be used to find species with significant differences between groups at each taxonomic level. (Figures 4A,B). ICGA400 treatments enhanced the Bacteroides (*p* < 0.05) in contrast to the CON group, while Proteobacteria (*p* < 0.01), Actinobacteriota (*p* < 0.05), Acidobacteriota (*p* < 0.05), Gemmatimonadota (*p* < 0.01), and Verrucomicrobiata (*p* < 0.05) decreased notably. Compared with the CON group, *Terrisporobacter* (*p* < 0.05), *Romboutsia* (*p* < 0.05), *Ruegeria* (*p* < 0.05), *Turicibacter* (*p* < 0.05), *Cutibacterium* (*p* < 0.05), and *Staphylococcus* (*p* < 0.05) showed a dramatic reduction in the ICGA400 group, while the *Megasphaera* (*p* < 0.01), *Catenibacterium* (*p* < 0.05), *Rikenellaceae_RC9_gut_group* (*p* < 0.05), *Selenomonas* (*p* < 0.05), *Lachnospiraceae_AC2044_group* (*p* < 0.05), and *Prevotellaceae_UCG-003* (*p* < 0.05) increased significantly.

## 4 Discussion

CGA and ICGA are natural phenolic compounds abundantly present in coffee, fruits, and vegetables. ICGA, also known as dicaffeoylquinic acids, predominantly include 3,5-dicaffeoylquinic acid (isochlorogenic acid A), 3,4-dicaffeoylquinic acid (isochlorogenic acid B), and 4,5-dicaffeoylquinic acid (isochlorogenic acid C) (20–22). Owing to their dietary prevalence and favorable biological functions, these compounds have attracted considerable interest and are extensively utilized in pharmaceuticals, food additives, and related fields (23, 24). However, research focusing on the use of ICGA in livestock and poultry production remains relatively limited compared to other disciplines, highlighting the need for further investigation into their potential applications in animal nutrition.

As natural bioactive compounds, chlorogenic acids (CGA) are known to exert antidiarrheal and growth-promoting effects when incorporated into animal diets. Numerous studies in broilers have reported that dietary CGA supplementation enhances growth performance, improves meat quality, and reduces the feed/gain ratio (F/G) (25–28). Similar benefits have been observed in monogastric animals, where CGA has been associated with improved growth and intestinal function (29–31). Notably, both CGA and isochlorogenic acids (ICGA) possess structurally similar phenolic profiles and exhibit comparable antioxidant, antibacterial, and anti-inflammatory activities. In the present study, dietary ICGA supplementation significantly increased the average daily gain (ADG) of weaned piglets, which is consistent with earlier findings on CGA. However, differences in absorption and metabolic pathways between CGA and ICGA may lead to distinct *in vivo* effects. We found that piglets receiving 200 or 400 mg/kg ICGA exhibited higher apparent digestibility of crude protein, gross energy, ether extract, and ash. This result aligns with previous reports that ICGAs can inhibit ɑ-glucosidase and ɑ-amylase (32, 33), suggesting a modulation of nutrient hydrolysis and absorption. In particular, the improved digestibility of ether extract (crude fat) may indicate enhanced lipid utilization, a phenomenon also reported in other models where bioactive compounds such as pectic polysaccharides inhibited intestinal lipid absorption and promoted fecal lipid excretion (34). Although the numerical reduction in diarrhea incidence did not reach statistical significance, factors including ICGA dosage, animal health, and environmental conditions may have influenced these outcomes. Further studies employing higher dosages or different delivery strategies may help clarify the antidiarrheal potential of ICGA in weaned piglets.

Weaning is widely recognized as a critical period for piglets, often leading to oxidative stress caused by an imbalance in pro-oxidant and antioxidant systems (35). Antioxidant indicators, including T-AOC, SOD, CAT, GSH-Px, and MDA, provide insights into an animal’s endogenous defenses against reactive oxygen species (ROS). Enhancing these antioxidant defenses can help alleviate oxidative stress (36). ICGA, a polyphenolic compound abundant in various food sources, has been reported to exhibit potent antioxidant effects (23, 37). Structural features, such as the multiple hydroxyl groups found in caffeoylquinic acid moieties, are thought to underlie ICGA’s capacity to scavenge ROS (38). Previous work suggests that the antioxidant activity of ICGA surpasses that of CGA, possibly due to a greater number of hydroxyl functional groups (22). Our findings indicate that dietary ICGA supplementation boosted serum T-AOC and CAT activities, consistent with earlier studies (39, 40). In the study of (20) revealed that ICGA isoforms can counter oxidative stress by scavenging ROS in Caco-2 cells treated with pro-inflammatory proteins, potentially through activation of the Nrf2-Keap1-ARE signaling pathway. While the present results underscore ICGA’s beneficial impact on antioxidant capacity in weaned piglets, knowledge remains limited regarding its exact molecular mechanisms in swine. Factors such as dosage, synergy with other dietary antioxidants, and variations in immune status may all contribute to the observed antioxidant response.

Recent research has highlighted that ICGA, a prominent dietary polyphenolic compound, exhibits diverse biological functions, notably immunomodulatory capabilities (21, 41). The immune system encompasses an intricate network of tissues, organs, specialized immune cells, and bioactive immune molecules (42). Immunoglobulins, as pivotal immune effectors, play essential roles within the humoral immune response, mediating the host’s defense against pathogenic invasion (43). In the present study, supplementation of ICGA significantly elevated serum IgA and IgM concentrations in weaned piglets at day 14 compared to the control (CON) group, reaching peak values at the dietary inclusion level of 400 mg/kg. Furthermore, on day 28, a significant enhancement in IgM concentration was observed in the serum of piglets receiving the highest dietary dose (800 mg/kg ICGA), relative to the CON group. The balance and interaction between pro-inflammatory and anti-inflammatory cytokines are crucial determinants of effective immune protection against pathogens (44). Numerous studies indicate that ICGA possesses marked anti-inflammatory, antimicrobial, and antiviral properties (5). However, in this investigation, dietary ICGA supplementation did not affect mucosal concentrations of sIgA or the cytokines IL-2, IL-4, IL-10, and IFN-*γ* in the jejunum and ileum of weaned piglets. It is important to note the scarcity of existing data specifically addressing the immunomodulatory effects of ICGA in piglets, and the current experimental conditions may not have presented a sufficient pathogenic or environmental challenge. Further exploration is therefore necessary to comprehensively evaluate the potential of ICGA to modulate immune function and confer protection against pathogenic threats in piglets.

The gut microbiota fundamentally regulates host metabolism, gastrointestinal function, and immune development (45), and serves as a key biomarker of intestinal health (46). Among external factors, diet exerts a dominant influence on the structure and function of the gut microbial community (47). Specific dietary components—including functional ingredients and feed additives—may improve growth performance and gastrointestinal health by modulating microbial composition and stability (48). In this study, however, ICGA supplementation did not significantly alter the *α*-diversity of colonic microbiota—as measured by observed_otus, Shannon, Simpson, Chao1, and Goods_coverage indices—across treatment groups. Notably, this absence of alpha-diversity modulation contrasts with reports in low-birth-weight (LBW) piglets, where a hydrolyzed protein formula significantly reduced microbial diversity indices (e.g., Chao1, ACE, Shannon, Simpson), yet enhanced barrier function and immune outcomes (49). This discrepancy may arise from differences in basal health status between normal and LBW models, or from distinct bioactivities of the supplements. Nevertheless, our results imply that ICGA may promote gastrointestinal homeostasis without disrupting microbial diversity, potentially sustaining a stable colonization niche that facilitates immune maturation—a particularly desirable effect in vulnerable populations such as LBW neonates.

The relative abundance histogram indicated that Firmicutes and Bacteroidetes were predominant bacterial phyla, aligning with prior findings (50). *Bacteroidetes* are recognized as highly competitive commensal organisms within the gut microbiota, significantly contributing to essential metabolic processes in the colon, particularly the fermentation of carbohydrates and metabolism of nitrogenous substrates (51). Major metabolic by-products generated by anaerobic fermentation in *Bacteroidetes* include acetic acid, succinic acid, and isovaleric acid, which serve as energy sources for the host (52). Additionally, these bacteria play a protective role by suppressing the colonization of pathogenic microorganisms in the gut ecosystem (53). In the present study, we observed that dietary ICGA significantly increased the abundance of the *Bacteroidetes* in the colonic digesta of piglets. A similar outcome was described before (54), who observed increased cecal *Bacteroidetes* abundance in piglets fed chlorogenic acid. The LEfSe, designed to detect biomarkers with significant differential abundance within microbial communities (55), indicated *Bacteroides*, *Prevotella*, and *Muribaculaceae* as dominant taxa in piglets from the ICGA400 treatment, whereas *Peptostreptococcus*, *Actinomycetes*, and *Proteobacteria* prevailed in the CON group. Prevotella possesses the ability to degrade plant-derived polysaccharides and host-derived mucins, generating beneficial metabolites such as short-chain fatty acids, including propionate (56). *Muribaculaceae*, a recently reclassified bacterial family formerly known as S24-7, belongs to *Bacteroidetes* and responds variably to dietary modifications and host physiological conditions, though its precise functional roles require further clarification (57). Alterations in the abundance of *Muribaculaceae* appear closely related to dietary interventions and host-specific physiological conditions; however, the precise functional roles and mechanisms of these bacteria remain poorly defined, necessitating further detailed investigations. Another notable observation from this study was the marked decrease in *Proteobacteria* abundance within cecal microbiota samples following ICGA supplementation. *Proteobacteria*, characterized as Gram-negative organisms possessing an outer membrane rich in lipopolysaccharides, represent a diverse and clinically significant bacterial phylum encompassing well-known pathogenic genera, such as *Escherichia* and *Helicobacter* (58). Elevated proportions of *Proteobacteria* are typically indicative of intestinal dysbiosis or host pathology, underscoring its potential as a microbial biomarker of compromised gut health (59). The beneficial effects of ICGA on growth performance and gut health in piglets may be attributed to its ability to modulate intestinal microbiota through probiotic-driven competitive exclusion. Probiotics potentially inhibit pathogen proliferation by competing for essential nutrients, colonization sites, and secreting antimicrobial metabolites (60). At the genus level, compared with the CON group, *Terrisporobacter*, *Romboutsia*, *Ruegeria*, *Turicibacter*, *Cutibacterium*, and *Staphylococcus* were significantly reduced in the ICGA400 group, while the *Megasphaera*, *Catenibacterium*, *Rikenellaceae_RC9_gut_group*, *Selenomonas*, *Lachnospiraceae_AC2044_group*, and *Prevotellaceae_UCG-003* increased significantly. These beneficial microbes are recognized producers of short-chain fatty acids, known to strengthen the antioxidant defense system and activate anti-inflammatory signaling pathways, thus maintaining intestinal homeostasis and overall health (61). Furthermore, ICGA supplementation can modulate the intestinal microbial ecosystem by effectively suppressing the proliferation of potential pathogens, thus promoting a balanced gut microbiota and enhancing overall intestinal health. Maintaining this microbial equilibrium is crucial in preventing dysbiosis-associated disorders and contributes to improved physiological resilience in animals.

## 5 Conclusion

Taken together, the present study demonstrated that dietary ICGA supplementation could modestly reduce post-weaning diarrhea, while effectively improve nutrient digestibility, antioxidant activity and humoral immune status of weaned piglets. ICGA modulated intestinal microbiota composition by increasing the abundance of beneficial microbiota and reducing the abundance of harmful bacteria in weaned pigs.

## Data Availability

The datasets for this study can be available on request to the corresponding author. The raw sequencing data are available from NCBI repository: https://www.ncbi.nlm.nih.gov/, under accession number PRJNA1330785.
